# Interplay between Transcription Factors and the Epigenome: Insight from the Role of RUNX1 in Leukemia

**DOI:** 10.3389/fimmu.2015.00499

**Published:** 2015-09-29

**Authors:** Kate H. Brettingham-Moore, Phillippa C. Taberlay, Adele F. Holloway

**Affiliations:** ^1^School of Medicine, University of Tasmania, Hobart, TAS, Australia; ^2^Genomics and Epigenetics Program, The Garvan Institute of Medical Research, Sydney, NSW, Australia

**Keywords:** epigenome, chromatin, cancer, epigenetic mechanisms, RUNX1

## Abstract

The genome has the ability to respond in a precise and co-ordinated manner to cellular signals. It achieves this through the concerted actions of transcription factors and the chromatin platform, which are targets of the signaling pathways. Our understanding of the molecular mechanisms through which transcription factors and the chromatin landscape each control gene activity has expanded dramatically over recent years, and attention has now turned to understanding the complex, multifaceted interplay between these regulatory layers in normal and disease states. It has become apparent that transcription factors as well as the components and modifiers of the epigenetic machinery are frequent targets of genomic alterations in cancer cells. Through the study of these factors, we can gain unique insight into the dynamic interplay between transcription factors and the epigenome, and how their dysregulation leads to aberrant gene expression programs in cancer. Here, we will highlight how these factors normally co-operate to establish and maintain the transcriptional and epigenetic landscape of cells, and how this is reprogramed in cancer, focusing on the RUNX1 transcription factor and oncogenic derivative RUNX1–ETO in leukemia as paradigms of transcriptional and epigenetic reprograming.

## Introduction

The gene expression profile of a cell determines its phenotype and function, and transcription factors play a key role in defining these gene expression profiles in response to cellular signals. Typically they do so by binding to *cis*-acting elements within promoters, enhancers, and other gene regulatory regions and functioning within regulatory complexes to control gene activity. However, control of gene expression is exerted by mechanisms involving the interaction of transcriptional complexes with not only the DNA sequence itself but also the chromatin proteins associated with the DNA. Access to the DNA by transcription factors is controlled by the packaging of DNA within the nucleus as chromatin. The specific composition of chromatin can dictate gene expression patterns in a cell by regulating the relative accessibility provided to transcription factors and the transcriptional machinery. Although the tight packaging of nucleosomes into heterochromatin inhibits transcription factor access, and transcription, euchromatin, with its associated relaxed chromatin and nucleosome positioning is more conducive to transcriptional activation due to the relative ease of transcription factor access. Chromatin composition is dynamic and is maintained or modified through the concerted actions of transcription factors and chromatin modifiers responding to cellular signaling cascades. Not surprisingly then, mutations to transcription factors and the molecules that comprise and modify the chromatin landscape commonly underlie the altered gene expression profiles that are characteristic of cancer cells. Herein, we will focus on the co-operative actions of these factors to determine the transcriptional and epigenetic landscape of cells, and how this is reprogramed by modifications to key regulatory factors in cancer.

## Mechanisms of Epigenetic Control

Within the eukaryotic cell, DNA is assembled into chromatin, consisting of repeating units of nucleosomes. Each nucleosome is a complex of eight histone proteins (two each of H2A, H2B, H3, H4) around which 147 base pairs of DNA are wrapped. This complex forms the basis of the epigenetic landscape of a cell, which in its broadest definition encompasses DNA methylation, histone modifications, histone variants, and nucleosome positioning (1). The epigenetic landscape in a given cell is therefore determined by the actions of an array of DNA and chromatin-modifying enzymes, chromatin remodeling complexes, recently identified chromatin-associated signaling kinases, as well as non-coding RNAs (2–6). Together, these are often referred to as epigenetic readers, writers and erasers (7). As the name suggests epigenetic writers add modifications to the DNA and chromatin, which alter gene expression capabilities, while erasers remove these modifications. Epigenetic readers recognize specific epigenetic modifications on DNA or histones and include chromatin remodeling enzymes, chromatin modifiers, chromatin architectural proteins, and adaptor proteins.

Perhaps the most widely investigated mechanism of epigenetic control is DNA methylation, the modification of DNA involving the addition of methyl groups to CpG residues, by DNA methyltransferases. CpG dinucleotides are found throughout the genome in dense clusters, referred to as CpG islands that are associated with a large proportion of gene promoters. CpG methylation is generally, although not always, associated with gene silencing. CpG methylation facilitates gene repression by acting as a recruiting surface for proteins, which possess methyl binding domains and initiate chromatin condensation. Interestingly, DNA methylation alone has been shown to further promote chromatin condensation (8). To a lesser extent, DNA methylation can also facilitate gene repression by physically blocking the binding of transcriptional regulators. DNA methylation was previously thought to be a highly stable modification that can only be removed following DNA replication, it has been recently recognized that intermediate forms exist that are thought to be precursors of the demethylated state (9). However, DNA methylation does not exist in isolation, with the associated histones normally reflecting the modified DNA in terms of active or repressive modifications.

Histone tails can be post-translationally modified via the addition or removal of numerous chemical groups, with over a dozen now reported, including acetyl, methyl, and phosphate groups (10). These modifications are reversible and the chromatin state that exists at any particular time is a result of the competing actions of, for example, histone acetyltransferases and histone deacetylases and methyltransferases competing with demethylases. Particular modifications can alter the biophysical composition of the chromatin or can act as recruitment surfaces for the epigenetic readers, thus leading to the idea of the histone code (7). However, it is becoming clear that histone modifications do not act in isolation, and it is instead the combination of modifications present at a particular region of DNA, which defines its functional outcome. The defining example of this is the colocation of repressive H3K27me3 and activating H3K4me3 modifications marking a “bivalent” chromatin state, which maintains certain genes in a primed state in embryonic stem cells (11).

Nucleosome structure can also be altered via the inclusion of histone variants within the histone octamer. During chromatin assembly and disassembly, histone variants can be deposited and exchanged by histone chaperones. Histone variants possess unique structural and functional properties. For example, the inclusion of the histone variant H3.3 is associated with transcriptionally active regions but is also deposited in telomeric and pericentric heterochromatin (12). The histone chaperones responsible for this histone exchange, nucleosome assembly and disassembly play a key role in controlling the chromatin nucleoprotein landscape.

Epigenetic plasticity is further regulated by the three-dimensional and higher-order chromatin structure, including nucleosome repositioning, DNA looping, and long-range chromatin interactions. Chromatin remodeling enzymes use the energy released from hydrolysis of ATP to reposition nucleosomes and include large multi-subunit complexes, such as SWI/SNF, ISWI, Nurd/Mi/CHD, SWR1, and INO80. These complexes must be recruited to the appropriate regulatory region to enable remodeling, which then facilitates access to the transcription factors and transcriptional machinery (13, 14). The spatial organization of chromatin plays a role in gene regulation with recent evidence demonstrating that certain inactive chromatin domains interact with the nuclear lamina, as reviewed in Ref. (15). At a more localized level, chromatin loops can form between regulatory regions in actively transcribed genes that are often hundreds of kilobases apart, with both *cis* and *trans* interactions possible (16, 17).

## Interplay between the Epigenome and Transcription Factors

The dynamic nature of the epigenome can be attributed to the interaction of transcription factors with chromatin and chromatin-modifying enzymes. Transcription factors must gain access to their binding sites within a chromatin context. However, once bound to DNA, transcription factors can also modify the chromatin landscape. Signaling pathways influence the chromatin landscape by activating transcription factors, which then bind to regulatory regions of DNA, recruiting with them chromatin modifying and remodeling enzymes. Signaling kinases can, however, also impact the chromatin landscape directly [reviewed in Ref. (18)]. For example, both the signaling kinases, PKC-theta (5) and ERK2 (19), have recently been found to have nuclear functions as chromatin-associated proteins. While it has long been known that protein kinases operate by communicating signals from the cytoplasm to the nucleus, it is also now evident that these and other nuclear kinases can also associate with chromatin in the nucleus impacting the chromatin landscape directly by phosphorylating histone proteins (2).

## Disruption of Transcription Factors and the Epigenome in Cancer

Dysregulation of the epigenetic landscape is evident in cancer with a variety of modifications observed in tumors. Aberrant DNA methylation, histone modifications and variant usage, non-coding RNA expression, and higher order chromatin structure have all been documented across a variety of cancer types (20–22). All of these modifications impact the chromatin profile and expression of the cancer genome. Not surprisingly then, mutations in transcription factors and epigenetic enzymes are frequently observed in cancer (23–25) with mutations characterized in numerous cancer types and in chromatin remodeling enzymes, histone modifiers, DNA methyltransferases, and non-coding RNA processing enzymes.

In terms of DNA methylation, cancer cells have a characteristic signature. Comparison of normal versus tumor tissue has demonstrated that cancer is typically characterized by global hypomethylation along with promoter specific hypermethylation (26, 27). Global hypomethylation is considered to promote genetic instability, fragile sites, and oncogene activation while hypermethylation in cancer is associated with repression of tumor suppressor gene transcription. There is also evidence for long-range epigenetic silencing (28). These abnormal methylation profiles can be driven by numerous factors including mutation in the enzymes responsible for distributing this epigenetic mark. In acute myeloid leukemia (AML), the DNMT3A enzyme is frequently mutated with approximately 20% of patients having some form of coding mutation (23).

Histone modifications in cancer have been less frequently studied, however, once again certain patterns are characteristic of cancer cells, for example profiling H4 revealed a decreased level of H4K16 acetylation and H4K20 trimethylation as an almost universal hallmark of cancer cells (20). Chromatin remodeling and modifying enzymes are also frequently mutated in cancer. SWI/SNF mutations have been detected at a frequency of 20% (24), with the majority of these being inactivating mutations, which points to a likely role as a tumor suppressor. Together, these alterations contribute to the epigenetic plasticity and dysregulation of cancer cells, a key property in terms of transformation events and the gene expression programs required for epithelial to mesenchymal transition.

Thus, in cancer cells signaling pathways, transcription factors and the chromatin regulatory networks are altered, resulting in transcriptional and epigenetic reprograming that ultimately drives increased proliferation and the hallmark features associated with neoplasia.

## RUNX1: A Paradigm of Transcriptional and Epigenetic Reprograming in Leukemia

A hallmark of leukemia is somatic mutations and genetic rearrangements that impact signal transduction and gene expression programs, with disruption to chromatin modifiers and transcription factors prevalent. A unique understanding of the dynamic interplay between transcription factors and the epigenome can be gained through study of such transcription factors, and particularly how alterations to the transcription factors lead to reprograming of transcriptional networks and the epigenome. The RUNX1 transcription factor and its oncogenic derivative RUNX1–ETO are paradigms of transcriptional and epigenetic reprograming in leukemia.

The RUNX1 transcription factor is a key regulator of hematopoiesis with its disruption resulting in abnormal hematopoiesis (29). *RUNX1* is frequently disrupted by genetic alterations in leukemia, and was originally identified following characterization of the *t*(8;21) chromosomal translocation found in AML. This translocation fuses the N-terminal region of the *RUNX1* gene to the *RUNX1T1* gene (also known as Eight-Twenty One or ETO), generating a RUNX1–ETO chimeric protein (30). While this is the most common chromosomal alteration to the *RUNX1* gene, up to ten other translocations have been found to disrupt this gene in leukemia (31). In addition, *RUNX1* loss-of-function mutations are associated with leukemia, as well as with Familial Platelet Disorder with propensity to develop AML (FPD/AML) (31).

**Figure 1:**
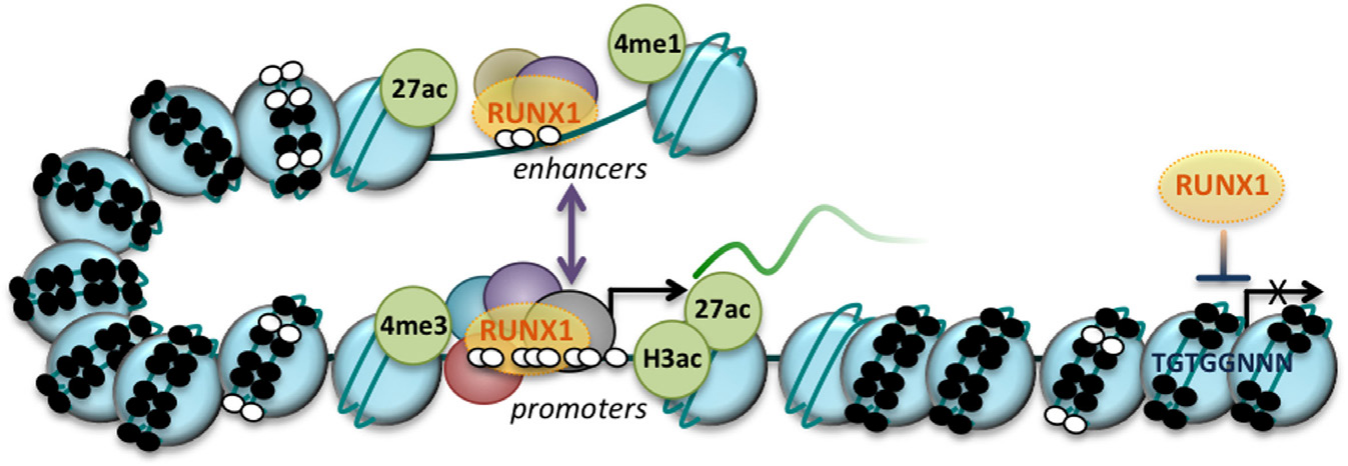
**RUNX1 regulates gene expression at both the transcriptional and epigenetic level**. RUNX1 binding to promoters and enhancers is regulated by the chromatin structure encompassing its binding sites (TGTGGNNN, as indicated), with condensed chromatin (nucleosomes, large blue circles) acting as a barrier to RUNX1 binding. DNA methylation (small black circles) may further inhibit RUNX1 binding. RUNX1 binds in a complex with other transcriptional regulators and coactivators/corepressors (represented by purple, red and green circles) at regions of open chromatin (nucleosome-depleted regions), which can impact gene expression through interactions with the transcription machinery or by modifying chromatin structure and composition. At enhancers, RUNX1 binding is accompanied by active histone marks, histone 3 lysine 27 acetylation (27ac), and histone 3 lysine 4 monomethylation (4me1) while at promoters, RUNX1 binding is associated with histone 3 lysine 3 trimethylation (4me3), histone 3 acetylation (of lysine 9 and 14; H3ac), and histone 3 lysine 27 acetylation (27ac). These active gene regulatory elements may interact through DNA loops (purple bidirectional arrow) and are devoid of DNA methylation (small white circles). Arrow, transcriptional start site (TSS).

While early studies characterized RUNX1 as a transcriptional activator, and demonstrated co-operation between RUNX1 and a range of other transcription factors to drive promoter activity (32, 33). RUNX1 has also been found to act as a transcriptional repressor in some circumstances (34). The effect of RUNX1 on target gene expression is thus highly context dependent, determined by the composition of the transcriptional complexes in which RUNX1 functions at a particular gene. These complexes contain transcriptional cofactors as well as epigenetic modifiers, which are recruited to gene promoters as well as other regulatory regions, and by doing so, RUNX1 can influence both transcriptional activity as well as the epigenetic status of target genes (Figure 1).

**Figure 2:**
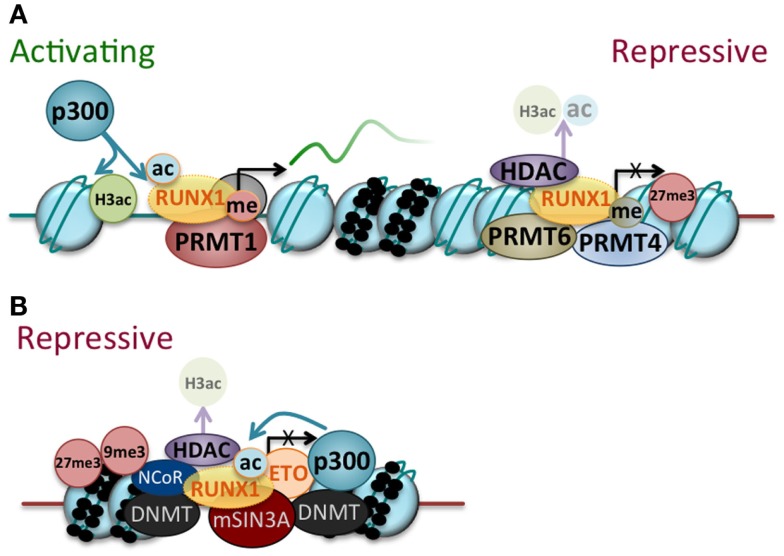
**RUNX1 and RUNX1–ETO regulate gene expression by recruiting transcriptional cofactors and epigenetic modifiers**. **(A)** RUNX1 can act as a transcriptional activator or repressor dependent on the balance of coactivators/corepressors associated with it at a particular time. RUNX1 can recruit coactivators [for example, p300 histone acetyltransferase (p300) and protein arginine methyltransferase (PRMT1)] and epigenetic modifiers, which enhance RUNX1 activity through post-translational modification (shown as acetylation (ac) and methylation (me) of the RUNX1 protein) and impart activating modifications to chromatin [for example, histone 3 acetylation of lysine 9 and 14 (H3ac)]. RUNX1 can also recruit corepressors and epigenetic modifiers [for example, PRMT6 and PRMT4 as well as histone deacetylases (HDAC)], which inhibit RUNX1 activity through post-translational modification (removal of acetylation marks from RUNX1 and chromatin) and establish repressive chromatin modifications (for example, histone 3 lysine 27 trimethylation, 27me3). Various continuums of the two extremes that are shown no doubt exist. **(B)** RUNX1–ETO binds to RUNX1 target genes and primarily acts as a transcriptional repressor through the assembly of repressive transcriptional and epigenetic complexes. While many of the molecules that associate with RUNX1–ETO are shown [for example, DNA methyltranferases (DNMT), nuclear receptor corepressor (NCoR), mSin3A corepressor complex (mSin3a), p300, and HDAC], they do not necessarily all complex with RUNX1–ETO at a given time. In keeping with the primarily repressive function of RUNX1–ETO, its binding is generally associated with repressive histone modifications (for example, histone 3 lysine 27 trimethylation (27me3) and histone 3 lysine 9 trimethylation (9me3).

RUNX1 has been found to complex with an array of epigenetic modifiers, with the outcome for both RUNX1 function and target gene activity dependent on the balance of activating and repressive factors associated with RUNX1 at a particular time (Figure 2A). RUNX1 interacts with the histone acetyltransferases, such as p300 and CBP, which potentiate RUNX1-dependent transcriptional activation of individual target genes (35, 36). More recently, elegant studies have demonstrated the orchestration of genome-wide changes by RUNX1 during hematopoietic development (37), with RUNX1 binding associated with increased histone acetylation. These studies illustrate the RUNX1-dependent recruitment of acetylating complexes to modify the epigenetic state of RUNX1 target genes.

RUNX1 itself is also a target of acetyltransferases as well as other modifiers, with modification of RUNX1 activity through interaction with epigenetic modifiers emerging as a common theme. Acetylation of RUNX1 by p300 was found to enhance its DNA-binding activity (38). Methylation of RUNX1 by the arginine methyltransferase PRMT1 increases RUNX1 transcriptional activity by disrupting its interactions with the msin3A corepressor complex (39). In contrast, methylation of RUNX1 by another arginine methyltransferase PRMT4 or CARM1 was found to initiate the formation of a RUNX1-dependent repressor complex (40). In addition, the histone methyltransferase SUV39H1 was found to interact with RUNX1 (41, 42), resulting in decreased DNA-binding activity and inhibiting RUNX1 activation of target gene promoters. The composition of the RUNX1 complexes can also be directly influenced by cellular signaling cascades with phosphorylation of RUNX1 by ERK also found to potentiate its transactivation activity by disruption its interactions with mSin3A (43, 44). Similarly, RUNX1 phosphorylation by cylin-dependent kinase (cdk) reduced its interaction with HDAC1 and HDAC3 and potentiated its transactivation activity (45).

These studies thus build a picture of RUNX1 itself as a target of epigenetic modifiers, which post-translationally modify the RUNX1 protein, altering its coactivator/corepressor interactions and thus influencing its transcriptional activity. In addition, RUNX1 recruits these coactivators and corepressors to target genes, resulting in modification of the chromatin environment and thus affecting gene activity at this second level. An elegant example of this multi-layered interaction between RUNX1, epigenetic modifiers and the epigenome is demonstrated in the recently described interaction of RUNX1 with protein arginine methyltransferase 6 (PRMT6). PRMT6 was found to be a component of a repressive RUNX1 complex also containing sin3a and HDAC1 (46). Recruitment of PRMT6 to target genes by RUNX1 resulted in asymmetric dimethylation of H3 (H3R2me2a). These genes were also found to be enriched for H3K27me3 and H3K4me2, but not H3K4me3. While these modifications were observed in hematopoietic progenitor cells, upon megakaryocyte differentiation, PRMT6 dissociated from the complex, which instead was found to contain the coactivators WRD5/MLL and p300/pCAF, resulting in loss of the H3R2me2a mark and enrichment for H3K4me3 and K3K9ac, which was associated with increased gene expression. In addition to such localized chromatin changes, RUNX1 has also been shown to initiate changes in higher order chromatin structure, mediating interactions between distal regulatory elements by facilitating chromatin looping (47).

The oncogenic RUNX1–ETO fusion protein contains the RUNX1 DNA binding domain but the transcriptionally active C-terminal domain of RUNX1 is replaced by almost the entire ETO protein. While RUNX1–ETO alone is not sufficient to induced leukemic transformation, it is sufficient to promote self-renewal and disrupt myeloid and erythroid differentiation (48). The RUNX1–ETO protein is proposed to bind to RUNX1 target genes, and genome-wide analysis has confirmed that RUNX1 and RUNX1–ETO binding largely overlap (49–51), however RUNX1–ETO is thought to primarily, although not exclusively, interact with co-repressor complexes causing repression of RUNX1 targets (Figure 2B). Interacting proteins include HDACs/N-CoR/msin3A, DNMT1, and p300 (52–57). In keeping with this, there are now multiple reports of epigenetic silencing of individual RUNX1 target genes by RUNX1–ETO associated with increased repressive chromatin modifications, such as H3K9 and H3K27 methylation (58–60). Similarly epigenetic reprograming on a genome-wide level by RUNX1–ETO has now also been described (50, 61). Interestingly, RUNX1–ETO was found to be recruited to regions of increased chromatin accessibility in association with p300, although this was not reflected in increased acetylation of the associated chromatin, but rather in the enrichment of repressive histone marks (62). p300 has been found to acetylate RUNX1–ETO and potentiate its activity (57), and the suggestion was that p300 may be able to acetylate RUNX1–ETO directly but not counteract the actions of repressive epigenetic modifiers also recruited by RUNX1–ETO on the chromatin platform. It has thus become apparent that RUNX1–ETO recruits different sets of epigenetic modifiers to target genes than RUNX1, directing reprograming of the epigenetic landscape and therefore resulting in altered gene expression profiles that contribute to the development of a leukemic state.

## Concluding Remarks

Over recent years, we have gained considerable insight into how chromatin structure is modified and the impact of this on the genome. Attention has now turned to understanding the dynamic and multifaceted interactions between transcription factors and epigenetic modifiers, and how they co-operate to determine gene expression responses to cellular signals. This is providing a better understanding of how alterations to these key regulatory molecules in cancer, as well as other disease processes, impact gene expression profiles. Further, this is offering hope that a better understanding of the interaction between aberrant transcription factors and the epigenome of cancer cells will provide increased opportunities for intervention in the disease. Interest in this field is being stimulated by the promising advances that are being made in cancer treatment with current epigenetic therapies, as highlighted recently (63). For example, the BET proteins, which recognize acetyl lysine modifications of histones are emerging as exciting therapeutic targets. A recent study demonstrated the potential of a specific inhibitor of BET proteins (iBET) to inhibit proliferation of myeloproliferative neoplasms driven by constitutively active Janus kinase 2 (JAK2) (64).

## Authors Contribution

KB conducted literature research and drafted sections of the manuscript and edited the manuscript. PT edited the manuscript and prepared figures. AH conducted literature research, drafted sections of the manuscript and edited the manuscript.

## Conflict of Interest Statement

The authors declare that the research was conducted in the absence of any commercial or financial relationships that could be construed as a potential conflict of interest.
